# The relationship between sex and functional outcome in first-episode schizophrenia: the role of premorbid adjustment and insight

**DOI:** 10.1017/S0033291723000442

**Published:** 2023-10

**Authors:** Joseph Ventura, Kenneth L. Subotnik, Sam Han, Gerhard S. Hellemann, Michael F. Green, Keith H. Nuechterlein

**Affiliations:** 1 UCLA Department of Psychiatry and Biobehavioral Sciences, Jane and Terry Semel Institute for Neuroscience and Human Behavior at UCLA, University of California, Los Angeles, California, USA; 2 Graduate School of Education and Psychology, Pepperdine University, Malibu, California, USA; 3 School of Public Health, Biostatistics Department, University of Alabama, Tuscaloosa, Alabama, USA; 4 VA Greater Los Angeles Healthcare System, Los Angeles, California, USA; 5 UCLA Department of Psychology, University of California, Los Angeles, California, USA

**Keywords:** Age of onset, first episode schizophrenia, functional outcome, insight, mediation analysis, neurocognition, premorbid adjustment, sex differences

## Abstract

**Background:**

Studies that examined sex differences in first-episode patients consistently show that males compared to females have poor premorbid adjustment, earlier age of onset, worse clinical characteristics, and poorer outcomes. However, little is known about potential mediators that could explain these sex differences.

**Methods:**

Our sample consisted of 137 individuals with first episode schizophrenia (males, *n* = 105; 77%) with a mean age of 22.1(s.d. = 4.1) years and mean education of 12.5(s.d. = 1.7) years. At entry, patients were within 2 years of their first psychotic episode onset. Baseline assessments were conducted for premorbid adjustment, symptoms, cognitive functioning, insight, and at 6-months for role and social functioning.

**Results:**

Males as compared to females had poorer premorbid adjustment across several key developmental periods (*p* < 0.01), an earlier age of onset [M = 20.3(3.3) *v.* 22.8(5.6), *p* = 0.002], more negative symptoms (*p* = 0.044), poorer insight (*p* = 0.031), and poorer baseline and 6-month role (*p* = 0.002) and social functioning (*p* = 0.034). Several of these variables in which males showed impairment were significant predictors of 6-month role and social functioning. Premorbid adjustment and insight mediated the relationship between sex and role and social functioning at 6-months, but not negative symptoms.

**Discussion:**

Males compared to females were at lower levels across several key premorbid and clinical domains which are strongly associated with functional outcome supporting the hypothesis that males might have a more disabling form of schizophrenia. The relationship between sex with role and social functioning was mediated through premorbid adjustment and insight suggesting pathways for understanding why females might have a less disabling form of schizophrenia.

## Introduction

The general conclusion from studies of multi-episode schizophrenia is that males compared to females appear to be at greater risk of developing schizophrenia and have a more severe form of the illness with regard to onset, symptoms, and psychosocial functional outcome (Abel, Drake, & Goldstein, 2010; Aleman, Kahn, & Selten, 2003). Understandably, researchers have turned to the initial course of schizophrenia to further examine early sources that contribute to sex differences. First episode studies also show that males compared with females have an earlier age of illness onset, lower levels of premorbid adjustment, lower baseline social and role functioning, more negative symptoms, and overall poorer course and outcome (Ochoa, Usall, Cobo, Labad, & Kulkarni, 2012). Researchers are interested in understanding sex differences in social and role functioning in search of ways to better understand prognosis and to improve outcomes.

Studies published thus far have generally not addressed possible mediation pathways that could explain better outcomes observed in females. This process involves examining the literature for possible mediators such as age of onset, premorbid adjustment, neurocognition and social cognition, and negative symptoms that have been found to been associated with outcomes in schizophrenia. Mediation analyses can then examine whether those predictors of outcome are mediator of the relationship between sex and functional outcomes. One study is an example of this approach, finding that females had better verbal memory compared to males and that a sequence of verbal memory and negative symptoms mediated functioning assessed at one year (Buck et al., 2020).

Better premorbid functioning in schizophrenia has been associated with a better prognosis, so sex differences in premorbid adjustment might have implications for short and long-term functional outcome (Addington & Addington, 2005; Brill et al., 2009; Minor et al., 2015; Stoffelmayr, Dillavou, & Hunter, 1983). One recent study suggested that the more favorable premorbid and baseline functional characteristics of women predicted better outcomes during the first three years of follow-up when treatment was delivered in early intervention services (EIS) (Ayesa-Arriola et al., 2020). Despite the age of onset and premorbid functional differences, generally these studies of first episode schizophrenia (FES) a term we use for brevity that includes individuals with schizophreniform and schizoaffective disorder, found few or no sex differences in demographic variables, most types of symptoms, and functioning at study entry. However, the findings are mixed for duration of untreated psychosis (Chang et al., 2011; Hui et al., 2016; Thorup et al., 2007, 2014). Given its prominent role, premorbid adjustment might be a potential mediator of the relationship between sex and functional outcome.

While there are few sex differences reported in positive symptoms, males reportedly have higher levels of negative symptoms while females show higher levels of mood symptoms such as depression and anxiety (Thorup et al., 2014). In fact, Cotton et al. (2009) found that women presented with higher levels of affective symptoms in general than did men. In contrast, Szymanski et al. (1995) found in individuals with schizophrenia admitted for the first time, that women compared to men presented with more anxiety, but also illogical thinking, inappropriate affect, and bizarre behavior. However, in one study no gender differences were found (Barajas, Ochoa, Obiols, & Lalucat-Jo, 2015). In any event, a review of FES studies concluded that males generally had more negative symptoms and females had more mood symptoms while there were little or no differences found in positive symptoms (Ochoa et al., 2012). Given the prominent role that negative symptoms play in functional outcome, negative symptoms might be a potential mediator of the relationship between sex and functional outcome.

The literature on cognitive deficits in FES is comparable with that on multi-episode patients showing that cognitive deficits are present (Mesholam-Gately, Giuliano, Goff, Faraone, & Seidman, 2009) and that there is a relationship between neurocognitive and social cognitive functioning and outcome in first episode patients (Fu, Czajkowski, Rund, & Torgalsbøen, 2017; Horan et al., 2012; Milev, 2005). Further, FES studies show that males perform better in the domain of visual perception while females perform better in domains such as verbal learning and memory (Fu et al., 2017; Hui et al., 2016; Pu et al., 2019; Torgalsbøen, Mohn, & Rund, 2014; Yang, Gao, Xiong, & Zhang, 2021). In one of the largest studies of sex differences in FES, late onset male patients were found to be more impaired than females on measures of verbal memory, executive functions, and other areas of cognitive performance (Ayesa-Arriola et al., 2020). Studies suggest that the superior social cognitive performance of females is not present in individuals with FES (Danaher, Allott, Killackey, Hester, & Cotton, 2018; Navarra-Ventura et al., 2018; Verdaguer-Rodríguez et al., 2021) for a review see Mote and Kring (2016). In the general population, women on average perform somewhat better on verbal tasks while men perform somewhat better on visuospatial tasks (Kern et al., 2008a, 2008b; Weiss et al., 2003). Several studies that examined neurocognitive deficits in schizophrenia did not include a healthy control group and/or did not correct for sex differences in the general population. Whether these effects are present in schizophrenia to a larger extent than in the general population is not clear (Lewine, Thurston-Snoha, & Ardery, 2006). Thus, whether the conclusions regarding sex differences in neurocognition for schizophrenia patients reflect the impact of schizophrenia or general population sex differences is difficult to determine.

There is still much to be learned about sex differences in insight for individuals with first episode psychotic disorders. In a prospective study using structured assessments, FES women as compared with men showed better insight into psychotic illness (McEvoy et al., 2006). However, additional studies of FES individuals that also used structured assessments did not find significant sex differences in global awareness of having a mental disorder (Ayesa-Arriola et al., 2011; Keshavan, Rabinowitz, DeSmedt, Harvey, & Schooler, 2004; Mutsatsa, Joyce, Hutton, & Barnes, 2006). The possibility that there are sex differences in insight early in the course of schizophrenia is important because a large body of evidence in schizophrenia links better insight with lower levels of symptoms and better functioning. Better insight among females, if present, could be a good prognostic sign and help explain the better course, outcomes, and recovery rates seen in females. Given the prominent role for insight in functional outcome, insight might be a potential mediator of the relationship between sex and functional outcome.

We hypothesized that FES males compared to females have poorer premorbid adjustment, an earlier age of onset, poorer insight into their illness, poorer baseline role and social functioning, and higher baseline levels of negative symptoms. Further, we planned to examine these variables as potential mediators of the relationship between sex and functional outcome to provide additional information regarding pathways by which sex influences early functional outcome in schizophrenia.

## Methods

### Participants

The patient sample consisted of 137 individuals with schizophrenia, schizophreniform, or schizoaffective disorder-depressed type, including 105 males (77%) and 32 females (23%). The mean age at study entry was 22.0 (s.d. = 4.1) years, the mean education was 12.5 (1.7) years, and most patients were single (*n* = 131; 86%). All participants received outpatient psychiatric treatment at the UCLA Aftercare Research Program and were participants in the fourth phase of the Developmental Processes in Schizophrenic Disorders Project (Nuechterlein et al., 2020; Subotnik et al., 2015). This study was approved by the UCLA Office for Human Research Protections, and all participants gave written informed consent. Diagnosis was established by trained and certified research assessment staff through the Structured Clinical Interview for DSM-IV (SCID-IV) plus supplementary information from family members and treating professionals.

Entry criteria were: (1) an onset of psychotic illness within 2 years of study entry; (2) a DSM-IV diagnosis of schizophrenia, schizoaffective disorder-depressed type, or schizophreniform disorder; (3) 18 to 45 years of age; (4) no evidence of a known neurological disorder; (5) no evidence of significant and habitual drug abuse or alcoholism in the 6 months prior to study entry and that the psychosis was substance-induced; (6) premorbid IQ not less than 70; (7) sufficient fluency in English to avoid invalidating research measures; and (8) treatment with risperidone was not contraindicated.

Normal comparison subjects were recruited through the use of flyers and advertisements which were placed in community sites and local newspapers. Normal comparison participants were chosen on the basis of age, sex, race/ethnicity, handedness, parental education, and community of residence that were comparable to those of the patient sample. Potential normal comparison subjects were assessed using the SCID-IV, sections of SCID-II for Personality Disorders, and the Brief Psychiatric Rating Scale (BPRS). The eligibility criteria included: no major psychiatric disorder except for a single episode of major depression, no personality disorder, no family history of a major psychotic disorder, and no current of substance use or history substance dependence.

### Procedures

Study Entry was defined as the time point in which the patient enrolled in the study. Baseline occurred about 2–3 months after study entry and the 6-month point occurred subsequent to the baseline assessment.

### Measures

#### Psychiatric history, premorbid adjustment, symptoms, and functioning

*UCLA Psychiatric and Social History Schedule (P&SH).* Demographic and premorbid history data were collected at study entry using a comprehensive form developed by the study team. P&SH data that were collected included age, sex, marital status, race, age of onset, patient and parental years of education, first appearance of prodromal and psychotic symptoms, and treatment history as well as additional psychiatric history variables.

*Cannon-Spoor Premorbid Adjustment Scale (PAS)* (Cannon-Spoor, Potkin, & Wyatt, 1982). The PAS domains include four developmental periods: (1) Childhood (up to age 11); (2) Early adolescence (ages 12 to 15); (3) Late adolescence (ages 17 to 18); and (4) Adulthood (age 19 and above). The PAS also includes five domains of psychosocial adjustment: (1) Sociability and withdrawal; (2) Peer relationships; (3) Scholastic performance; (4) Adaptation to school; and (e) Social-sexual aspects of life. A semi-structured interview accompanies the PAS items which are rated from 0 to 6 based on an interview with the patient and from all sources of information, e.g., the patient's parents, and school and medical records. The PAS was administered at baseline. We examined the summary scores for developmental periods for this study. Higher scores indicate more severe developmental delay.

*Brief Psychiatric Rating Scale (BPRS)* (Ventura, Green, Shaner, & Liberman, 1993a) The BPRS was used to examine severity levels in positive, negative, depression-anxiety, and manic symptoms (Ventura et al., 1993b). Each BPRS rater achieved a median Intraclass Correlation Coefficient (ICC) of 0.80 or higher across all BPRS items compared with a set of criterion ratings and participated in a quality assurance program. The BPRS is separated into factors (Ventura, Nuechterlein, Subotnik, Gutkind, & Gilbert, 2000) that include: **Positive Symptoms** (Unusual Thought Content, Hallucinations, and Conceptual Disorganization), **Negative Symptoms (**Blunted Affect, Emotional Withdrawal, and Motor Retardation), and **Depression-Anxiety** (Depression, Anxiety, Guilt). The BPRS ratings were administered at Study Entry.

*Scale for the Assessment of Negative Symptoms (SANS)* (Andreasen, 1984). The SANS is a 25-item measure that is widely used to assess two negative symptom domains: (1) Expressive Symptoms, which consisted of Affective Flattening (blunted affect) and Alogia, and (2) Experiential Symptoms, which consisted of Avolition/Apathy and Anhedonia/Asociality. The SANS was administered every 3 months by raters who were trained to criterion levels of ICC = 0.75 or higher on either the Global Items or all SANS items (Ventura et al., 1993a).

*Global Functioning Scale: Role (GFS: Role) and Global Functioning Scale: Social (GFS: Social)* (Cornblatt et al., 2007; Niendam, Bearden, Johnson, & Cannon, 2006). The GFS: Role is a 10-point rating scale that measures a combination of the quantity and quality of work/school functioning. The GFS: Social is a 10-point rating scale that measures a combination of the quantity and quality of social interactions with family and friends. Both versions of the GFS ratings were completed at study entry and at 6 months based on their ongoing interactions with the patients and family members. Raters were individual therapists who were trained raters, with mean ICC of ICC = 0.91 for GRS: Role and ICC = 0.76 for the GFS: Social.

### Neurocognition and social cognition

*MATRICS Consensus Cognitive Battery (MCCB)* was administered at Baseline to provide a standardized assessment of cognitive functioning (Nuechterlein et al., 2008). This battery assesses seven domains: Speed of Processing, Attention/Vigilance, Working Memory, Verbal Learning, Visual Learning, Reasoning and Problem Solving, and Social Cognition. The dependent variable was the MCCB Overall Composite Score and the individual domain scores for which we used a scaled T scores based on a community sample (Kern et al., 2008b). Due to the focus on sex differences, we did not use T scores corrected for gender and age.

*The Awareness of Social Inference Test (TASIT)* Part III uses video clips depicted individuals either lying or expressing sarcasm (McDonald, Flanagan, Rollins, & Kinch, 2003). After each item (video clip and hint), the participants were asked four questions meant to gauge their ability to make inferences about the characters' actions, intentions, thoughts and feelings. A total score was calculated across lie and sarcasm items (Kern et al., 2008a).

*Relationships Across Domains (RAD)* is a 75-item paper-and-pencil assessment of an individual's perception of various aspects of relationships. The RAD has good internal consistency in schizophrenia patients and normal controls (Sergi et al., 2009). The RAD reflects the ability of individuals to use their implicit knowledge of four relational models to comprehend social relationships and infer the behavior of social partners. The RAD contains 25 vignettes. After each vignette, participants were asked yes-no questions that evaluate their implicit knowledge of the relational model. The dependent measure was the total score across the four relational models.

*Mayer-Salovey-Caruso Emotional Intelligence Test 2.0 (MSCEIT)* is a self-report measure consisting of 141 items and 8 subscales that evaluate four components (branches) of emotion processing (Mayer, Salovey, & Caruso, 2002). The MSCEIT has been shown to demonstrate good reliability and discriminant validity in schizophrenia (Eack et al., 2010; Eack, Greeno, Christian-Michaels, Dennis, & Anderson, 2009; Kee et al., 2009). The MISCEIT contains four branches: Identifying Emotions, Using Emotions, Understanding Emotions, and Managing Emotions. In this study we used a global measure from the MSCEIT, Emotional IQ (EIQ).

### Insight

*Scale to Assess Unawareness of Mental Disorder (SUMD)* (Amador, Strauss, Yale, & Gorman, 1993) is a multi-dimensional scale based on a semi-structured interview. The SUMD items and subscales define specific components of insight – General Unawareness of (Having a) Mental Disorder, Unawareness of the Achieved Effects of Medication, Unawareness of Symptoms, Misattribution of Symptoms, and Unawareness of the Social Consequences of (Having a) Mental Disorder. To reduce the number of relationships examined, we chose one primary variable to approximate overall insight, i.e., General Unawareness of (Having a) Mental Disorder. This primary item represents an individual's lack of understanding that the symptoms or experiences of the prior psychotic episode at study entry were part of a mental illness. SUMD items are scored from 1 (aware) to 5 (unaware) so higher scores indicate greater unawareness. Additional components of unawareness were examined in secondary analyses after the primary variable of general unawareness of mental disorders was analyzed for a sex difference.

### Statistical approach

We conducted *t* test, ANOVA, and Chi-square analyses to examine sex differences between male and female first-episode schizophrenia patients on variables collected during Study Entry, Baseline, and at 6 months after baseline.

**Fig. 1.**
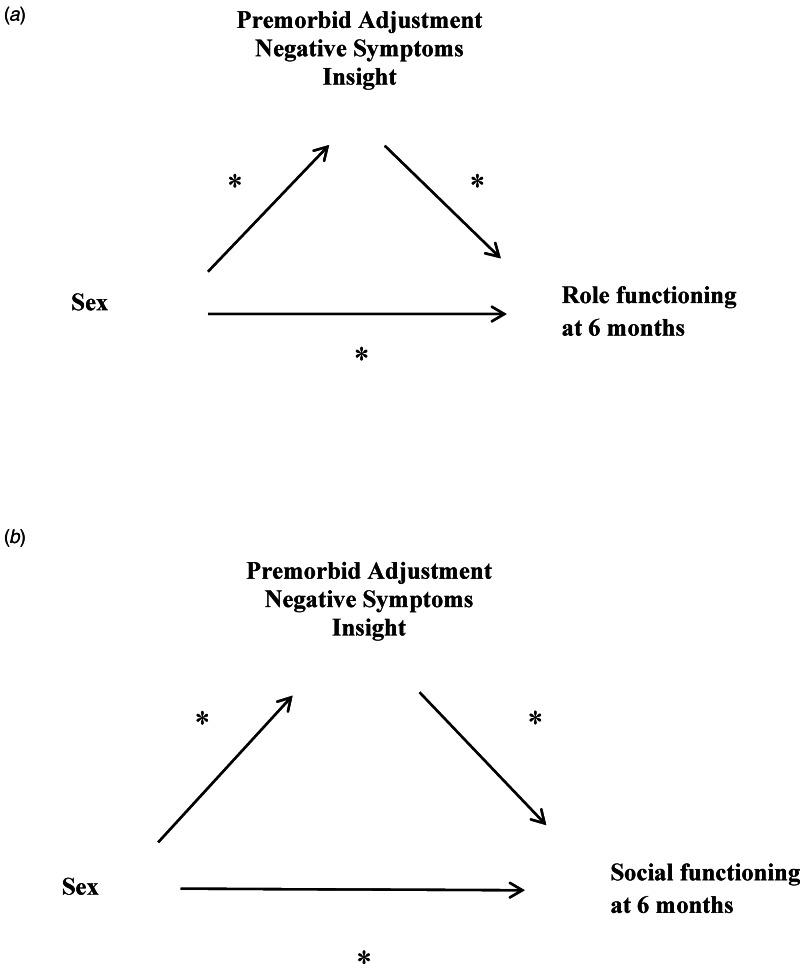
(a) Model one examines role functioning at 6 months. Examination of potential mediators of the relationship between sex and role functioning (school and work) at 6-months using a boot strapping method to generate confidence intervals. (b) Model two examines social functioning at 6 months. Examination of potential mediators of the relationship between sex and social functioning (family and friends) at 6-months using a boot strapping method to generate confidence intervals.

There were significant relationships among several potential predictor variables such as premorbid adjustment, negative symptoms, and insight measured at baseline in relationship to role and social functioning at 6 months. Mediation analyses (Model One) were used to evaluate whether the relationship between sex and role functioning (school and work) was mediated by these predictor variables (Fig. 1a). We also examined (Model Two) the indirect effect of premorbid adjustment, negative symptoms, or insight on the relationship between sex and social functioning (friends and family) (Fig.1b). Analyses were conducted on functioning at the 6-month point. We followed the well-established procedures and conceptual understanding for mediation provided by Baron and Kenny (1986) and using bootstrap confidence intervals for the indirect effects of the mediators (Preacher & Hayes, 2004).

## Results

Demographic information and a statistical analysis of sample characteristics can be found in Table 1 which contains a list of variables, means, standard deviations, *F* or *t* test values, and *p* values.

**Table 1.** Sex differences in demographic, premorbid adjustment, clinical, symptom, and insight variables in individuals with first episode schizophrenia, total *n* = 137

Variables of interest	Total sample mean (s.d.) %	Males (*n* = 105)	Females (*n* = 32)	*F*, *t*, or χ^2^ Statistic	*p* value
Age at study entry	22.0 (4.1)	21.5 (3.2)	23.8 (5.7)	8.45	0.004
Education	12.5 (1.7)	12.4 (1.6)	12.8 (1.9)	1.57	0.212
Marital status	Single 133 (96%) Married 4 (4%)	104 (99%) 1 (1%)	29 (91%) 3 (9%)	χ^2^ 4.94	0.026
Premorbid adjustment^a^					
Childhood	1.40 (0.95)	1.52 (0.94)	1.03 (0.92)	6.13	0.015
Early adolescence	1.64 (0.96)	1.76 (0.94)	1.28 (0.93)	5.67	0.019
Late adolescence	1.91 (1.0)	2.05 (1.0)	1.48 (1.0)	6.90	0.010
Adulthood	2.32 (1.4)	2.54 (1.4)	1.56 (1.2)	8.90	0.004
Total premorbid adjustment score	1.85 (0.84)	2.01 (0.81)	1.34 (0.76)	13.6	0.000
Age of onset^b^	20.9 (4.1)	20.3 (3.3)	22.8 (5.6)	9.52	0.002
Duration of psychotic symptoms^b^ (months)	8.11 (8.7)	8.57 (9.1)	6.42 (6.7)	1.24	0.266
Total time Ill (including prodrome)^b^ (months)	12.18 (11.1)	12.73 (10.8)	10.14 (12.2)	1.02	0.315
Duration of untreated psychosis (DUP)^b^	20.1 (22.5)	18.5 (20.4)	24.6 (27.2)	1.63	0.204
BPRS symptoms^c^					
Negative	2.28 (1.0)	2.38 (1.1)	1.94 (0.9)	4.11	0.044
Positive	3.60 (1.5)	3.57 (1.5)	3.69 (1.5)	0.16	0.684
Mood	2.63 (1.2)	2.57 (1.2)	2.81 (1.3)	0.80	0.372
SANS negative symtoms^d^					
Expressive	2.04 (1.1)	2.17 (1.1)	1.60 (1.1)	4.72	0.032
Experiential	2.89 (1.0)	3.00 (0.9)	2.54 (1.2)	3.57	0.062
SANS Total	2.48 (0.9)	2.58 (0.9)	2.08 (1.1)	5.40	0.022
Global daily functioning^e^					
Role (work and school) baseline	4.21 (2.2)	3.85 (2.0)	5.35 (2.4)	9.66	0.002
Role six months	5.27 (2.5)	4.75 (2.4)	6.95 (2.0)	13.0	<0.001
Social (friends and family) baseline	5.95 (1.8)	5.75 (1.8)	6.62 (1.6)	4.58	0.034
Social six months	6.32 (1.8)	6.09 (1.9)	7.05 (1.6)	4.07	0.047
Insight into mental disorder (SUMD)^f^					
General unawareness of (having a) mental disorder	2.62 (1.5)	2.81 (1.5)	2.00 (1.2)	5.76	0.018
Unawareness of the benefits of medication	2.25 (1.5)	2.47 (1.6)	1.58 (0.98)	7.07	0.009
Unawareness of past delusions	3.75 (2.0)	4.04 (2.1)	2.92 (1.3)	5.98	0.016
Misattribution of past delusions	2.82 (1.9)	3.06 (2.0)	2.04 (1.5)	5.49	0.080
Unawareness of past hallucinations	4.23 (2.6)	4.43 (2.6)	2.60 (2.7)	1.81	0.181
Misattribution of past hallucinations	2.99 (2.6)	3.13 (2.6)	2.56 (2.8)	0.84	0.361
Unawareness of the social consequences (of having) a mental disorder	1.90 (1.5)	2.09 (1.6)	1.29 (0.9)	5.16	0.025

a Cannon-Spoor Premorbid Adjustment Scale.

b Psychiatric and Social History Form.

c Brief Psychiatric Rating Scale (BPRS).

d Scale for the Assessment of Negative Symptoms (SANS)

e Global Functioning Scale: Social and Role.

f Scale to assess unawareness of mental disorder; for GFS analyses at 6 months, total *n* = 84, *n* = 60 males; *n* = 24 females.

We found that males compared to females had lower levels of premorbid adjustment across the four key developmental periods including Childhood (*p* = 0.015), Early Adolescence (*p* = 0.019), Late Adolescence (*p* = 0.010), and Adulthood (*p* = 0.004), and had an earlier age of onset, M = 21.7 (s.d. = 3.2) *v.* 23.7 (s.d. = 4.8), *p* = 0.004 (Table 1). In keeping with the analyses of premorbid adjustment, we found at study entry that males were rated lower than females in Role Functioning (*p* = 0.002) and Social Functioning (*p* = 0.034) and at 6 months (Table 1). Symptom assessments at study entry indicated that males as compared to females exhibited higher levels of negative symptoms (*p* = 0.044), but that there were no differences between the two groups in positive symptoms (*p* = 0.684) or mood symptoms (*p* = 0.372). In addition, we found that at baseline females had better insight than males (*p* = 0.018; Table 1) in that they scored lower on Unawareness of Having a Mental Disorder. Also, secondary analyses revealed that females were significantly lower (better insight) than males on Unawareness of the Benefits of Medication, Unawareness of Past Delusions, and Unawareness of the Social Consequences of Having a Mental Disorder (Table 1).

Because sex differences in some cognitive domains tend to be present in the general population, we examined cognitive functioning on the MCCB at baseline through Diagnosis (Schizophrenia *v.* Healthy Comparison) × Sex (Male *v.* Female) ANOVAs (Table 2). We found no significant Diagnosis × Sex interactions for the Overall Composite Score or any domain score. Further, there were no statistically significant Diagnosis × Sex interactions at baseline on any of the measures of social cognition. As expected, both male and female patients performed more poorly compared to their respective control group.

**Table 2.** Examination of sex differences in neurocognition and social cognition in individuals with first episode schizophrenia compared with healthy controls *T* score means and s.d.

Variables of interest	Patients total sample Mean (s.d.)	Patients males Mean (s.d.)	Patients females Mean (s.d.)	Healthy controls total sample Mean (s.d.)	Control males Mean (s.d.)	Control females Mean (s.d.)	*F* ratio/*p* value for Group x sex interaction
*Neurocognition*							
MCCB^a^ overall							
Composite	43.52 (14.1)	43.0 (13.5)	44.59 (15.5)	52.5 (10.3)	52.0 (10.5)	53.2 (10.2)	449 / 0.50
Speed of processing	39.5 (11.2)	39.8 (11.4)	38.5 (11.1)	53.7 (9.4)	53.2 (9.3)	54.5 (9.7)	0.54 / 0.46
Attention/Vigilance	38.7 (11.6)	39.2 (11.2)	37.4 (12.9)	51.7 (9.7)	52.2 (9.5)	50.9 (10.1)	0.01 / 0.90
Working memory	46.5 (13.4)	47.7 (13.2)	42.9 (13.7)	52.5 (11.5)	53.1 (12.0)	51.7 (10.9)	0.69 / 0.40
Verbal learning	39.6 (9.6)	39.7 (8.9)	39.4 (11.0)	50.8 (9.9)	49.7 (9.5)	52.6 (10.3)	0.97 / 0.32
Visual learning	44.3 (10.4)	44.9 (10.4)	42.7 (10.4)	49.9 (9.8)	49.3 (11.0)	51.0 (7.8)	1.35 / 0.24
Reasoning problem							
Solving	47.3 (8.9)	48.4 (8.8)	44.1 (8.5)	51.7 (9.4)	52.3 (9.0)	50.8 (10.1)	0.89 / 0.34
Social cognition	37.3 (12.9)	37.1 (12.7)	37.8 (14.0)	51.5 (8.9)	50.4 (9.1)	53.2 (8.4)	0.29 /.58
*Social cognition*							
TASIT^b^	22.0 (4.2)	22.3 (5.2)	21.0 (4.1)	25.9 (3.7)	26.9 (4.0)	24.5 (2.7)	0.374 / 0.54
RAD^c^	47.8 (8.8)	47.5 (9.1)	48.8 (8.0)	59.8 (5.2)	60.1 (4.6)	59.3 (6/1)	0.564 / 0.45
MSCEIT^d^	88.2 (15.2)	88.1 (15.1)	88.3 (15.8)	100.3(12.1)	101.6 (12.5)	98.2 (11.5)	0.462 / 0.50

a MATRICS Consensus Cognitive Battery (MCCB) *T* scores are uncorrected for age and gender.

b The Awareness of Social Inference Test (TASIT).

c Relationships Across Domains (RAD).

d Mayer-Salovey-Caruso Emotional Intelligence Test 2.0 (MSCEIT); Patients: MCCB *n* = 98, TASIT *n* = 82; RAD; *n* = 83; MSCEIT *n* = 86; Controls: MCCB *n* = 92, TASIT *n* = 39, RAD *n* = 52, MSCEIT *n* = 45.

Given the significant sex differences in premorbid adjustment, and baseline negative symptoms and insight we conducted correlational analyses which indicated that better total premorbid adjustment was predictive of better role and social functioning at 6 months (Table 3). Lower negative symptoms as measured by the SANS were correlated with higher role functioning at 6 months and higher social functioning at 6 months (Table 3). In addition, correlational analyses indicated that better insight into having a mental illness at baseline was predictive of better role and social functioning 6 months later.

**Table 3.** Examination of baseline predictors of 3 month and 6 month role and social functioning in individuals with first episode schizophrenia (*n* = 84)

	Role and social functioning			
	Baseline		6 Month	
	Role	Social	Role	Social
Premorbid adjustment^1^				
Childhood	−0.18+	−0.15	−0.33**	−0.06
Early adolescence	−0.16	−0.29**	−0.32**	−0.16
Late adolescence	−0.12	−0.29**	−0.28*	−0.25*
Adulthood	−0.29*	−0.22*	−0.37**	−0.37**
Premorbid adjustment (Total)	−0.25*	−0.32**	−0.47**	−0.32**
Age of onset	0.15	0.08	0.13	0.17
Negative symptoms (at baseline)				
BPRS negative symptoms	−0.21*	−0.32**	−0.10	−0.13
SANS expressive	−0.22*	−0.49**	−0.28*	−0.43**
SANS experiential	−0.45**	−0.64**	−0.36**	−0.52**
SANS total	−0.38**	−0.64**	−0.37**	−0.55**
Insight at study entry				
General unawareness (of having) a mental disorder	−0.14+	−0.28**	−0.38*	−0.45**
Unawareness of benefits of medication	−0.18	−0.23*	−0.21*	−0.34**
Unawareness of past delusions	−0.11	−0.30*	−0.28*	−0.34*
Unawareness of the social consequences (of having) a mental disorder	−0.15	−0.29*	−0.28*	−0.19+

*p < 0.05; ** *p* < 0.01; + *p* < 0.10; *n* = 60 males; *n* = 24 females.

### Mediation analysis

We conducted analyses to determine if the relationship between sex and role functioning (work or school) at 6 months was mediated by any of three potential mediators: premorbid adjustment, negative symptoms, and insight (see Fig. 1 for model). The mediation path (indirect effect) through premorbid adjustment was significant (95% CI 0.22–1.4). After taking the mediation path through premorbid adjustment into account, sex does still have a direct effect on the outcome (95% CI 0.42–2.94) so premorbid adjustment is considered a partial mediator. The mediation path (indirect effect) through insight was significant (95% CI 0.00–0.74). After taking the mediation path through insight into account, sex does still have a direct effect on the outcome (95% CI 0.80–3.22) so insight was considered a partial mediator. However, the mediation path (indirect effect) through negative symptoms was not significant (95% CI −0.17 to 0.76).

We also conducted analyses to determine if the relationship between sex and social functioning at 6 months was mediated by any of the same three potential mediators (see Fig. 1b for model). The mediation path (indirect effect) through premorbid adjustment was significant (95% CI 0.02–0.86). After taking the mediation path through premorbid adjustment into account, sex does not have a direct effect on the outcome (95% CI −0.040 to 2.09) so premorbid adjustment is a full mediator. The mediation path (indirect effect) of insight was significant (95% CI 0.074–0.927). After taking the mediation path through insight into account, sex does not have a direct effect on the social functioning (95% CI −0.29 to 1.52) so insight is considered a full mediator. However, the mediation path (indirect effect) through negative symptoms was not significant (95% CI −0.15 to 0.96).

## Discussion

Our analyses are consistent with prior studies showing robust sex differences in a rigorously defined cohort of FES patients across several developmental, clinical, insight, and functional domains. Male individuals compared to females had lower levels of premorbid adjustment, an earlier age of onset, higher levels of negative symptoms, and lower levels of social and work functioning. In contrast, there were no sex differences in positive or mood symptoms. At study entry, females had better insight into having a mental illness and better insight into the benefits of medication and the social consequences of a disorder. Differences in premorbid adjustment, negative symptoms, and insight significantly predicted role and social functioning at 6 months for the entire sample. One key finding of our study is that premorbid adjustment and insight were mediators of the relationship between sex and functional outcome (role and social), while negative symptoms were not. Specifically, the mediation analyses indicate that pathways from the patient's sex to role and social functioning (school and work) go through premorbid adjustment and insight, suggesting that higher levels of functioning observed in females can partially be explained by the influence of sex on premorbid adjustment and insight. Although both males and females with schizophrenia performed more poorly than did healthy controls on measures of neurocognition and social cognition, no statistically significant diagnosis by sex interactions were found in cognitive performance.

The important role of premorbid functioning is consistent with prior studies which show an association with a better prognosis in first episode patients (Albert et al., 2011; Amminger et al., 2011). In addition, most studies have found that sex differences in premorbid functioning predict better functioning in women compared to men up to 3 years (Ayesa-Arriola et al., 2020). In addition, individuals with schizophrenia with higher levels of premorbid functioning generally demonstrate better treatment response to medications compared to individuals with lower levels (Hatzimanolis et al., 2020; Klein & Rosen, 1973; Rabinowitz et al., 2011; Strous et al., 2004).

Males typically have an earlier age of onset (Immonen, Jaaskelainen, Korpela, & Miettunen, 2017; Miettunen, Immonen, McGrath, Isohanni, & Jääskeläinen, 2019; Neill et al., 2020) and this finding has been extended to first episode patients, for a review see Ochoa (Ochoa et al., 2012). That is consistent with the view that males have a more severe form of the illness compared to females (Abel et al., 2010; Immonen et al., 2017; Rabinowitz, Levine, & Hafner, 2006). Indeed, the earlier the onset of symptoms, the less time an individual has to develop socially or academically before functional impairment occurs due to schizophrenia.

The observation of a relationship in our sample between greater baseline insight and higher role and social functioning at 6-months suggests that insight is an important predictor of the early course of schizophrenia. The finding that insight is a mediator of the relationship between premorbid adjustment and role functioning is important because there are sex differences in insight early in the course of schizophrenia and better insight is linked to lower levels of symptoms and better functioning (Cobo et al., 2016, 2020; Pousa et al., 2017; Ramu, Kolliakou, Sanyal, Patel, & Stewart, 2019). Insight into one's illness indicates the ability to reflect on one's experiences, which is likely related to the ability to understand the experiences of others as well. These Theory of Mind skills are related to better insight (Subotnik et al., 2020). Thus, the better awareness of a mental disorder in females at baseline might help to explain their better course and functional outcome. The implication is that EIS for people with schizophrenia might focus more attention for males on the development of insight, as this may lead to better functional outcome.

Broader issues in sex-related cultural stereotypes in expectations of males compared to females might interact with the clinical mediators we identified to influence the course of the illness. Congruent with gender stereotype theory, adolescent men may feel greater pressure to leave home and begin a career at an earlier age than do females (Hentschel, Heilman, & Peus, 2019). Also, several investigators have hypothesized that females tend to possess stereotypically female gender traits, such as acceptance of their illness and treatment adherence, while males tend to exhibit more socially adverse and self-destructive behaviors and attitudes (Morgan, Castle, & Jablensky, 2008; Riecher-Rössler & Häfner, 2000). The greater insight into having a mental illness that we found in females may represent an aspect of this acceptance of illness. On a related topic, whether socioeconomic or cultural influences on gender identity are a stronger influence than sex at birth on premorbid adjustment and insight into illness needs more investigation (Lewine et al., 2006).

Biological factors may also act as a mediator which could be an additional influence on the more favorable course observed in females compared with males with schizophrenia. In fact, the level of estrogen has been suggested as a protective factor (da Silva & Ravindran, 2015; Falkenberg & Tracy, 2014; Gogos et al., 2015; Hafner et al., 1994; Salem & Kring, 1998). The estrogen hypothesis postulates that the vulnerability threshold for schizophrenia is mediated by the estrogen levels in women until menopause (Abel et al., 2010; Gogos et al., 2015; Olsen et al., 2008). Estrogen is known to protect against prenatal complications and excessive synaptic pruning and may also have a antipsychotic-like effect by impacting post-synaptic dopaminergic signal transduction and reducing dopamine-mediated psychotic symptoms (Seeman, 2012). Hence, the modulating role of estrogen may be an additional factor in the later onset, better role and social functioning, and lower levels of negative symptoms observed in the early phase of illness.

We did not find any Diagnosis by Sex interactions in neurocognitive or social cognitive performance. Some prior studies that found sex differences in cognition among schizophrenia patients did not include a healthy control group and did not use cognitive scores that were corrected for sex differences in the general population. Thus, many of the prior reports of sex differences in cognitive performance among schizophrenia patients might be due to general population differences rather than to the impact of schizophrenia.

The study had some limitations. Some patients may not accurately recall how long ago their psychotic symptoms began. We believe that our use of parental and professional reports as well as medical records to minimize this issue. As is the case with many studies of individuals with schizophrenia, our sample size for females is smaller than for males. However, we were able to detect significant sex differences with this sample size of females. Because we received most of our referrals from hospitals at which patients were acutely ill, sample selection may favor those patients who required a hospitalization. Men or women who never needed hospitalization may therefore be underrepresented. Our sample consists of individuals who are willing to participate in clinical research. However, how these factors would differentially impact participation of males *v.* females to produce the sex differences we found is not obvious.

To summarize, in examining sex differences between males and female individuals with schizophrenia, we found many elements of consistency with the prior literature which indicates that males have poorer premorbid adjustment, an earlier age of onset, higher negative symptoms, and lower levels of role and social functioning. We found that predictors of functioning, such as premorbid adjustment and insight partially mediated the relationship between sex and role functioning and fully mediated the relationship between sex and social functioning. These findings underscore that males are more impaired than females and have prognostic implications for the impact of sex on the early course of schizophrenia.
